# Early detection of type 2 diabetes in socioeconomically disadvantaged areas in Stockholm – comparing reach of community and facility-based screening

**DOI:** 10.1080/16549716.2020.1795439

**Published:** 2020-08-04

**Authors:** Linda Timm, Katri Harcke, Ida Karlsson, Kristi Sidney Annerstedt, Helle Mölsted Alvesson, Nouha Saleh Stattin, Birger C Forsberg, Claes-Göran Östenson, Meena Daivadanam

**Affiliations:** a Department of Global Public Health, Karolinska Institutet, Stockholm, Sweden; b Academic Primary Health Care Centre, Region Stockholm, Stockholm, Sweden; c Department of Neurobiology, Care Sciences and Society, Karolinska Institutet, Stockholm, Sweden; d Department of Molecular Medicine and Surgery, Endocrine and Diabetes Unit, Karolinska Institutet, Karolinska University Hospital, Solna, Sweden; e Department of Food Studies, Nutrition and Dietetics, Uppsala University, Uppsala, Sweden; f International Maternal and Child Health Division, Department of Women’s and Children’s Health, Uppsala University, Uppsala, Sweden

**Keywords:** Community screening, facility-based screening, opportunistic screening, prediabetes, T2D, FINDRISC, hard-to-reach

## Abstract

**Background:**

Type 2 diabetes and its high-risk stage, prediabetes, are often undiagnosed. Early detection of these conditions is of importance to avoid organ complications due to the metabolic disturbances associated with diabetes. Diabetes screening can detect persons unaware of diabetes risk and the elevated glucose levels can potentially be reversed through lifestyle modification and medication. There are mainly two approaches to diabetes screening: opportunistic facility-based screening at health facilities and community screening.

**Objective:**

To determine the difference in population reach and participant characteristics between community- and facility-based screening for detection of type 2 diabetes and persons at high risk of developing diabetes.

**Methods:**

Finnish diabetes risk score (FINDRISC) is a risk assessment tool used by two diabetes projects to conduct community- and facility-based screenings in disadvantaged suburbs of Stockholm. In this study, descriptive and limited inferential statistics were carried out analyzing data from 2,564 FINDRISC forms from four study areas. Community- and facility-based screening was compared in terms of participant characteristics and with population data from the respective areas to determine their reach.

**Results:**

Our study found that persons born in Africa and Asia were reached through community screening to a higher extent than with facility-based screening, while persons born in Sweden and other European countries were reached more often by facility-based screening. Also, younger persons were reached more frequently through community screening compared with facility-based screening. Both types of screening reached more women than men.

**Conclusion:**

Community-based screening and facility-based screening were complementary methods in reaching different population groups at high risk of developing type 2 diabetes. Community screening in particular reached more hard-to-reach groups with unfavorable risk profiles, making it a critical strategy for T2D prevention. More men should be recruited to intervention studies and screening initiatives to achieve a gender balance.

## Background

Undiagnosed diabetes accounts for 50% of all cases globally [1] and one in three cases in Sweden [2], many persons are therefore unaware of their diabetes risk. The diabetes prevalence in Sweden is higher in non-European persons than Swedish born [3,4], thus areas with high proportions of foreign-born citizens have a higher prevalence of type 2 diabetes (T2D) (9.2% versus 5.4%) [5,6]. Screening has the potential to reach persons before symptoms appear [7,8]. American Diabetes Association (ADA) guidelines recommend screening for T2D and prediabetes [9], an established high-risk state [10]. In Sweden, screening for diabetes is recommended for persons with other factors conferring a high risk of developing T2D such as overweight, age and family history of diabetes, and follow-ups are recommended for persons with prediabetes and T2D [11]. Different tools exist for screening of high risk for T2D, such as diabetes risk scores like FINDRISC [12] or blood glucose measurements, such as glycosylated haemoglobin A1c (HbA1c), fasting glucose and oral glucose tolerance test (OGTT), which also provide biochemical confirmation of diabetes and prediabetes status [7,8,13]. Research has shown that participants experience diabetes screening as positive for risk awareness [14,15] and the average age for receiving a diabetes diagnosis has been shown to be 4.6 years lower for those who were screening-detected compared with clinically detected [16].

There are mainly two types of screening; opportunistic screening and open screening which is often community-based. Opportunistic screening, also called facility-based screening, is mostly done at health facilities such as primary health care centers (PHCC). These facility-based screenings seem most efficient because those who are found to have prediabetes are more likely to receive care [7]. Opportunistic screening initiatives at health facilities can also be more practical since the clinics have access to patient records and are equipped with the tests and personnel needed to conduct screening [17]. Community screening is an approach in which screening is offered to persons outside ordinary health care settings and has shown to be effective in reaching individuals who are at a higher risk of developing diabetes [18–21]. Community-based screening as opposed to facility-based screening has the potential to target persons who do not seek care actively [22,23]. Persons in low-socioeconomic strata and in ethnic minority groups have a higher risk of developing T2D [22] and their use of care in health facilities has been reported as low [22,24–26]. Migrants (defined as living in Europe and born outside their country of residence) have been shown to be less likely to utilize opportunistic screening services at health facilities than native-born [25], particularly when low socioeconomic status is the main factor influencing access to health services [24,25]. The utility and effectiveness of screening depend on its reach, the effectiveness of the screening tools and its intended outcome in terms of early detection of T2D, which in turn results in timely care and improved awareness among persons at high risk of developing T2D.

Reach is an aspect that affects both utility and effectiveness and is commonly defined as ‘the absolute number, proportion, and representativeness of individuals who are willing to participate in a given initiative’ [27]. Reach is determined by many factors, such as socio-economic status, level of education and unemployment and the degree of social isolation [18]. Disadvantaged populations are often described as hard-to-reach for health interventions [28]. These population groups have a higher risk for many non-communicable diseases including T2D, and at times facility-based screening strategies do not reach them adequately [4]. Additional barriers for attending screenings include previous poor experience of screening, worry about the procedure and result, lack of understanding about the purpose of screening, lack of time and perceived low risk of disease [29,30]. Interaction with the personnel conducting the screening and information received about the disease can encourage persons to be more positive to future screening [30,31].

To the best of our knowledge, no studies have compared the reach of community- and facility-based screening as part of T2D prevention. The aim of this study was to determine the difference in population reach and participant characteristics between community- and facility-based screening for detection of high risk of diabetes or T2D. Specifically, two research questions are investigated from an implementation perspective: 1) Who is reached through community- and facility-based screening when compared with eligible population from the study areas? 2) What are the participant characteristics related to diabetes risk scoring between community- and facility-based screening?

## Methods

This study is nested in two projects; SMART2D (Self-management approach and reciprocal learning for type 2 diabetes) and 4D (Four diagnoses: Diabetes Type 2, arthritis, breast cancer, and heart failure). SMART2D is a multi-country diabetes project based in Sweden, South Africa and Uganda funded by the EU Horizon 2020 Framework Program [32] targeting under-resourced or disadvantaged areas in three study sites: a rural area in Uganda, an urban township in South Africa and socioeconomically disadvantaged suburbs in Sweden. Screening in Sweden was done as part of both community engagement and study participant recruitment for the SMART2D adaptive implementation trial [33]. The 4D project was a collaboration between Karolinska Institutet and the Region Stockholm between 2013 and 2017, also located in socioeconomically disadvantaged areas. The 4D project aimed to map and improve the diabetes care process and to screen for prediabetes and T2D in primary care with the purpose of subsequent diabetes prevention [34].

**Figure 1.**
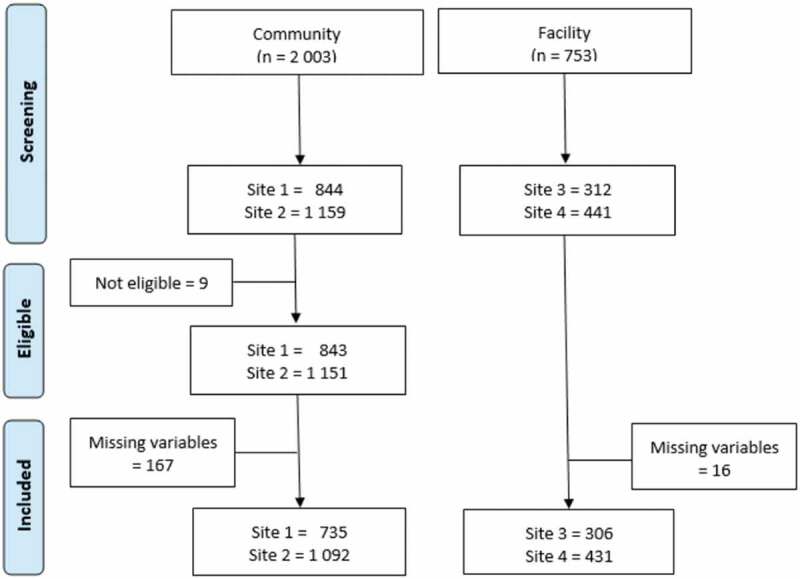
Prisma flow diagram showing participants in the two screening processes.

In total the SMART2D and 4D screenings resulted in data from 2,756 participants. Out of these, 192 participants were dropped from the analysis due to missing data concerning study site, sex, FINDRISC score, body mass index (BMI), place of birth or due to being under 18 years old (Figure 1) [35]. The analysis was thus conducted on 2,564 participants (community screening: 1827; facility-based screening: 737).

### Study setting

This study was conducted in four socioeconomically disadvantaged suburbs of Stockholm. The suburbs were characterized by low income levels, low educational levels and high unemployment rates. Most of the areas had a high proportion of migrants and a relatively high mobility as compared to other areas of the Stockholm region [36]. The Care Need Index (CNI), is a social deprivation index that uses socio-demographic variables to construct a composite score used as an indicator to assess need of care in a population [37]. The variables used in the construction of the CNI score include: Number of persons aged over 65 living alone; born abroad (Eastern Europe, Asia, Africa and South America); unemployed (aged 16–64); single parents with children 17 years or younger; persons who moved into the area (1 year or older); persons with low education (aged 25–64); children younger than five years. In the present study, the CNI was higher in these areas compared to more affluent suburbs in Sweden (Table 1).

**Table 1.** Study setting characteristics.

Study area	Site 1	Site 2	Site 3	Site 4
Total population, eligible for screening (age 18–75) Site 1 & 2: Year 2017 Site 3 & 4: Year 2014	18,814	37,549	27,027	13,292
Proportion of immigrants (persons born outside Sweden) Site 1 & 2: Year 2017 Site 3 & 4: Year 2014	60%	32%	36%	55%
Care Need Index (CNI) (all ages included) Year 2017	2.39	1.78	1.52	1.96

### Data collection process

#### Screening tool

FINDRISC is a validated tool used to identify individuals at high risk of developing diabetes or to detect asymptomatic T2D, consisting of eight questions about risk factors of diabetes [12,38]. It has been considered to be the most accurate and ideal tool to detect diabetes risk [38] and different cut-offs with acceptable sensitivity and specificity to detect diabetes risk have been identified for different population groups [39–41]. FINDRISC has been used in similar contexts in Sweden [3] and it has also been shown to be a useful tool in community screening [42]. Questions/measurements included are: age, BMI, waist circumference, level of physical activity, consumption of vegetables, fruits and berries, history of antihypertensive drug treatment, history of high blood glucose values and family history of diabetes. Each of the eight questions was scored between 1 and 5 points adding up to a total of 26 points in the commonly used version and 25 points in the modified version used by both SMART2D and 4D project. A cut-off point at ≥12 was defined as having a high risk of developing diabetes in order to reach as many persons as possible at high risk. In this study these persons are called high-risk participants and together they are called the high-risk group. In addition to the data collected through FINDRISC, the SMART2D and 4D projects collected information about each participant and her/his parents’ place of birth.

#### Community- based screening conducted by SMART2D project

Community screening took place between March 2017 and June 2018. In the preparation phase the SMART2D team had planning meetings at the participating primary health care centres, citizen service offices and local organisations at the two study sites. Employees from the citizen services offices helped mobilize people to the screening venues. Materials, such as leaflets and brochures, were designed and developed with information and health messages in simple Swedish language with a focus on illustrative pictures. Specially designed flags and roll-up posters were displayed at the venues to increase visibility. Most persons were screened in shopping malls, where many persons from the local communities were passing by daily. The screening was open to all adults who wanted to get screened. Other places targeted were local organisations, associations, women’s centres and swimming facilities. The screenings were conducted at different times throughout the day and the entire week including weekends. The screening consisted of conducting a FINDRISC questionnaire and a point of care HbA1 c test for those who scored above the predefined cut-off level in FINDRISC for a high risk of developing diabetes (≥12/25). The majority of responses were based on measurements conducted with the help of the screening team. A few responses were self-reported if participants were unwilling to carry out these measurements at the venue.

When a person had HbA1 c levels above 38 mmol/mol, they were either referred to one of the participating PHCCs through a written notification (if they were registered there) or recommended to go to a PHCC of their own choice for further examinations. This study only uses the data collected through the FINDRISC questionnaire.

#### Facility-based screening conducted by 4D project

The 4D project was carried out in two different phases. The first phase took place in two socioeconomically disadvantaged suburbs in Stockholm at the PHCCs between 2014 and 2015. The second phase was conducted in 15 different PHCCs in the Stockholm area in 2016 and 2017. In this study only the results from the first phase will be used since the questions added on participants’ backgrounds were not asked in the second phase. The information on the first phase screening was displayed on television-monitors at PHCCs. Participants for the study were recruited to the 4D project from the waiting rooms by an assistant nurse who helped them fill out the FINDRISC forms and conduct other tests for recruitment into the 4D study. Participants were also referred to screening by doctors and other personnel from the PHCC when they had an appointment at the health care centre for other health-related issues.

### Statistical analysis

Software program STATA 15.1 was used to analyze the data.

The analysis consists of two parts: 1) Comparing the reach of community and facility screening with respect to the eligible population living in the study areas. 2) Comparing the participant characteristics of the screened sample in community- and facility-based screening in terms of the FINDRISC variables and place of birth.

#### Comparison of reach

For the purpose of this study we have assessed reach as the ‘proportion, and representativeness of individuals who are willing to participate in a given initiative’ (community- and facility-based screening in this case) [27]. The sample population was compared against eligible population living in the study areas. For this purpose, we accessed the population data generated from the database at Statistics Sweden (SCB) [43]. Aggregated variables were purchased for the characteristics age, sex and place of birth for the four study sites corresponding to the same time period of the respective screening events. The population data from SCB were compared with the sample population on the above characteristics to understand who were reached through the different screening methods. The screening data were compared with population data on age, sex and place of birth, using the equality of proportions test to see if there were any differences between the two groups.

#### Comparison of participant characteristics

Descriptive and limited inferential statistics were performed to analyze the variables included in the FINDRISC tool (age, BMI, waist circumference, level of physical activity, consumption of vegetables, fruits and berries, history of antihypertensive drug treatment, history of high blood glucose values and family history of diabetes) (Table 3). To further analyze differences in terms of FINDRISC variables between community- and facility-based screening, Chi-square tests for proportions and Wilcoxon rank sum test for medians were used to compare whether the differences between the two groups were significant. Persons who were identified as high-risk participants (FINDRISC ≥12) were also analyzed separately using the same methods (Appendix, Table S1).

**Table 2.** Comparison of proportions & representativeness for the two screening methods with respect to the eligible population.

Study site	Community screening compared with Facility-based screening			Community screening compared with eligible target population from the study areas			Facility-based screening compared with eligible target population from the study areas		
	Community (%)	Facility (%)	P-value	Community (%)	SCB Com. (%)	P-value	Facility (%)	SCB Fac. (%)	P-value
Age groups									
Total (n)	1822	737	Chi2	1822	41979	Pr-test	737	31368	Pr-test
18–24	91 (5)	19 (3)	0.007	91 (5)	5315 (13)	< 0.001	19 (3)	4034 (13)	< 0.001
25–64	1461 (80)	522 (71)	< 0.001	1461 (80)	29539 (70)	< 0.001	522 (71)	21467 (68)	0.099
65–79	235 (13)	196 (27)	< 0.001	235 (13)	5575 (13)	0.861	196 (27)	4460 (14)	< 0.001
80–89	35 (2)	0 (0)		35 (2)	1550 (4)	< 0.001	0 (0)	1407 (4)	< 0.001
Sex									
Total (n)	1827	737		1827	56363		737	40319	
Female	1030 (56)	416 (56)	0.975	1030 (56)	27852 (49)	< 0.001	416 (56)	20059 (50)	< 0.001
Male	797 (44)	321 (44)	0.975	797 (44)	28511 (51)	< 0.001	321 (44)	20260 (50)	< 0.001
Place of birth									
Total (n)	1827	737		1827	56363		737	40319	
Africa	564 (31)	22 (3)	< 0.001	564 (31)	6126 (11)	< 0.001	22 (3)	2218 (6)	< 0.001
Asia	706 (39)	183 (25)	< 0.001	706 (39)	9031 (16)	< 0.001	183 (25)	7405 (18)	< 0.001
N. & C. America	6 (0)	5 (1)	0.220	6 (0)	308 (1)	< 0.001	5 (1)	218 (1)	< 0.001
S. America	56 (3)	46 (6)	< 0.001	56 (3)	1009 (2)	< 0.001	46 (6)	1067 (3)	< 0.001
Europe	132 (7)	76 (10)	0.010	132 (7)	6844 (12)	< 0.001	76 (10)	5962 (15)	< 0.001
Only Sweden	363 (20)	405 (55)	< 0.001	363 (20)	32934 (58)	< 0.001	405 (55)	23359 (58)	0.093
Unknown					91 (0)			90 (0)	

Abbreviations: SCB Com: Population eligible for screening from the community screening sites SCB Fac.: Population eligible for screening from the facility-based screening sites SCB: Statistics Sweden.

**Table 3.** Comparison of FINDRISC characteristics between community and facility-based screening.

Variables	Total (N = 2564)	Community (n = 1827)	Facility (n = 737)	P-value ^a^
Age median (IQR)	50 (38–61)	48 (37–59)	56 (43–65)	< 0.001
	n (%)	n (%)	n (%)	
Sex Female Male	1446 (56) 1118 (44)	1030 (56) 797 (44)	416 (56) 321 (44)	0.975
BMI ≤25 >25	890 (35) 1674 (65)	692 (38) 1135 (62)	198 (27) 539 (73)	< 0.001
Waist Women ≤88 >88 Men ≤102 >102	480 (33) 966 (67) 701 (63) 417 (37)	370 (36) 660 (64) 556 (70) 241 (30)	110 (26) 306 (74) 145 (45) 176 (55)	0.001 < 0.001
Physical activity 30 min/day Yes No	1787 (70) 777 (30)	1271 (70) 556 (30)	516 (70) 221 (30)	0.824
Intake of vegetables or fruit every day Yes No	1671 (65) 893 (35)	1221 (67) 606 (33)	450 (61) 287 (39)	0.005
Medication for high blood pressure Yes No	606 (24) 1958 (76)	383 (21) 1444 (79)	223 (30) 514 (70)	< 0.001
Elevated glucose values earlier in life Yes No	485 (19) 2079 (81)	315 (17) 1512 (83)	170 (23) 567 (77)	0.001
Family history of diabetes Yes No	1412 (55) 1152 (45)	964 (53) 863 (47)	448 (61) 289 (39)	< 0.001
Born in Europe Yes No	989 (39) 1575 (61)	503 (28) 1324 (72)	486 (66) 251 (34)	< 0.001
FINDRISC score <12 ≥12	1348 (53) 1216 (47)	1048 (57) 779 (43)	300 (41) 437 (59)	< 0.001
FINDRISC score median (IQR)	11 (8–14)	10 (7–14)	12 (9–16)	< 0.001^b^

^a^ = P-value is calculated using Chi-squared tests for proportions, except those marked^b.^

^b^ = P-value is calculated using Wilcoxon rank sum test of equality of medians.

## Results

Table 2 presents a comparison between community screening and facility-based screening and a comparison of data from the study samples and the target population, to assess the reach of the two screening methods. The table is described in terms of age groups, sex and place of birth and therefore shows the variation in representativeness of the sample population with respect to these variables, compared to data from SCB on the eligible population living in these areas. In comparison with background data of persons living in the study areas, persons born in Africa and Asia were to a higher extent reached through community screening compared with facility-based screening (p < 0.001). The opposite was true for persons born in Sweden and other European countries. Significantly less European born persons were reached at the community screenings compared with the population data. The community-based screening reached significantly more people at lower ages (age group 25–64 years), while the facility-based screening significantly reached older persons (age group 65–79 years). In general, women were over-represented in both types of screening (Table 2).

Table 3 presents the comparison of participants’ FINDRISC chracteristics between community- and facility-based screenings. The gender distribution was identical in the two screening groups but not the age distribution. Median age was 56 years in the facility-based screening compared with 48 in the community screening group. The participants at the facility-based screening were significantly more overweight, reported having taken medication for high blood pressure and have had known high glucose values at some time point earlier in life. Family history of diabetes was also reported to a higher degree. Reported physical activity did not differ between the groups. The median FINDRISC score was 10 for participants in the community screening and 12 among participants screened at the facilities (Table 3).

A further comparison between only high-risk participants screened in the communities versus the facilities showed that, overall, all characteristics became more similar between the two groups, including median FINDRISC scores and median age of participants (Table S1).

## Discussion

Community- and facility-based screening was utilised by different segments of the population. European and Swedish born participants were screened more frequently at the primary care facilities, while those with a non-European background, particularly from Asian and African countries, were screened more frequently at the community screening sites. This indicates differences in access to primary care for different population groups and that some segments will not be reached if only one method is used. Migrants, newly arrived residents, persons living in vulnerable social and economic situations are often seen as hard-to-reach populations [28]. Given the higher prevalence of T2D among foreign-born [3,4,44], and that migrants are not reached by health services to the same extent as native-born [25], it is unlikely that facility-based screening will reach as many in need as community screening. This is in line with other studies showing that hard-to-reach populations can be reached through community screening [18,19].

While the facility-based screening conducted at the PHCCs reached more high-risk participants (FINDRISC ≥12), those with an established genetic risk profile in terms of ethnicity were mainly reached through community screening. Persons born in Africa and Asia have a higher risk of developing diabetes due to genetic predisposition [45,46]. The prevalence of diabetes is also higher in general in African and Asian countries [47,48], and with the increase of migration from non-European countries the prevalence will most likely rise [44]. Stress and migration are associated factors [49], as well as stress and diabetes [50–52]. Therefore, it is particularly important to reach populations with a higher risk profile to detect potentially undetected T2D and prevent the disease from developing.

In this paper we discuss two individual high-risk approaches, that aim to give the screened participants information about their diabetes risk status and potential T2D, together with opportunities for lifestyle modification to reverse glucose levels. Other population-wide approaches, such as structural-level interventions to improve the social determinants of health in disadvantaged populations; or changes in environment to nudge people towards healthier lifestyle choices could potentially decrease the risk of developing diabetes. Thus, to minimize the risk of developing diabetes, especially for the persons at high risk, a combination of individualistic and population-wide approaches is most likely needed.

There are advantages and challenges of diabetes screening. Screening provides information that can lead to awareness of elevated glucose values and gives an opportunity to reverse or reduce the risk of developing diabetes [53]. In addition, complications of T2D such as retinopathy and cardiovascular disease [54,55] may be avoided through early diagnosis. It can therefore be argued that attention now should be given to how to screen instead of whether to screen or not. It is particularly important to screen for risk factors among those at highest risk for diabetes prevention [7]. An argument against screening for diabetes risk is harm caused by overdiagnosis in terms of giving unwarranted diagnosis without knowing whether it will result in manifest disease and suffering [56]. The risk awareness achieved through screening can potentially prolong the experience of the actual period of illness [57,58]. Prediabetes and diabetes risk are perceived as more abstract concepts compared with diagnosis such as T2D, and the information received through screening can be misunderstood or not interpreted as it was intended [59].

Optimal cut-offs for FINDRISC could also differ within the same country depending on the condition being screened (high risk, undiagnosed T2D or metabolic syndrome); the setting (population-wide or clinical) and the target population (men and women or different ethnic groups) [42,60–63]. This can lead to uncertainty and confusion. The ability of a screening test to identify high-risk individuals depends on its sensitivity and specificity. Cut-offs of 11–14 have been found to have acceptable sensitivity and specificity for FINDRISC in different populations [64,65]. However, this could mean higher proportion of the general population receiving a high score when overall prevalence and risk of developing T2D are low in that population [64]. Since the diabetes risk and prevalence in our study population are higher than the general population, it can be argued that our screening methods should have a higher precision in identifying those at high risk of developing T2D. A study in Sweden also recommended that Middle Eastern ethnicity be considered as an independent risk factor for T2D, instead of a change in FINDRISC cut-off point [3]. In this study, a FINDRISC cut-off of ≥12 was used to detect high risk of developing diabetes.

With respect to our study participants, in general we found that more high-risk participants were screened at PHCCs compared with the community screening. This is to be expected as participants who access facilities are already seeking care for other conditions or accompanying family members, often with a similar risk profile due to shared genetic, social and environmental factors. Although, the facility-based screening sample in this study could have led to selection bias, since only persons who signed consents to participate in the 4D project were scored using the FINDRISC tool. We also found that women accessed both screening methods more than men, and this is similar to other studies [66,67]. Also, women in general tend to be recruited to participate in intervention studies more often than men [68]. Therefore, efforts should be made to recruit more men to intervention studies and screening initiatives to achieve a gender balance.

In the community-based screening 43% of persons had high risk of diabetes according to FINDRISC (Table 3). Of these, 23% had no family history of diabetes and 64% had no history of elevated glucose levels earlier in life (Table S1). Thus, one could argue that a large proportion of the people found with high risk in the community could have been unaware of their risk status and would still be unaware if they had not been detected during the community-based screening. A similar pattern was seen among the high-risk participants in the facility-based screening. However, this group was already in contact with the primary care unit and had a greater chance of receiving information and advice from the health care staff during their visits, which is an argument for the need of community screening to reach the persons not reached by the health care system for early detection and treatment.

## Conclusion

We found that community-based screening and facility-based screening are accessed by different population groups with some overlap. Thus, both screening methods are needed to reach persons at high risk of developing T2D. Our study found that it is particularly important to implement screening in socioeconomically disadvantaged areas where the prevalence of diabetes and diabetes risk is higher than in the general population. Community screening in particular reached more hard-to-reach groups with unfavorable risk profiles, making it a critical strategy for T2D prevention.

## Supplementary Material

Supplemental Material

## Paper context

Diabetes screening is needed for early detection of T2D and persons with high risk of developing diabetes. This study investigates reach of screening and compares two screening methods, community- and facility-based. Our study shows that different segments of the population are reached through the two screening methods and therefore both are needed. It is particularly important to implement community-based diabetes screening in socioeconomically disadvantaged areas where diabetes risk and T2D prevalence is highest.
