# Supplementary material for: Early detection of type 2 diabetes in socioeconomically disadvantaged areas in Stockholm – comparing reach of community and facility-based screening
Source: Glob Health Action. 2020 Aug 4;13(1):1795439. doi: 10.1080/16549716.2020.1795439 (PMC7480601; doi:10.1080/16549716.2020.1795439)
Supplement: Supplemental Material [file ZGHA_A_1795439_SM6806.docx]

**Appendix**

**Table S1: High risk participants among all screened (FINDRISC scores ≥12)**

| **Variables** | **Total**  **(N=1216)** | **Community**  **(n=779)** | **Facility**  **(n=437)** | **P-value** ^a^ |
| --- | --- | --- | --- | --- |
|  | n (%) | n (%) | n (%) |  |
| **Sex**  Female  Male | 769 (63)  447 (37) | 512 (66)  267 (34) | 257 (59)  180 (41) | 0.016 |
| **BMI**  ≤25  >25 | 184 (15)  1032 (85) | 128 (16)  651 (84) | 56 (13)  381 (87) | 0.091 |
| **Waist**  Women  ≤88  >88  Men  ≤102  >102 | 127 (16)  642 (84)  169 (38)  278 (62) | 90 (18)  422 (82)  129 (48)  138 (52) | 37 (14)  220 (86)  40 (22)  140 (78) | 0.262  < 0.001 |
| **Physical activity 30 min/day**  Yes  No | 703 (58)  513 (42) | 435 (56)  344 (44) | 628 (61)  169 (39) | 0.063 |
| **Intake of vegetables or fruit every day**  Yes  No | 768 (63)  448 (37) | 521 (67)  258 (33) | 247 (57)  190 (43) | < 0.001 |
| **High blood pressure**  Yes  No | 456 (38)  760 (62) | 279 (36)  500 (64) | 177 (41)  260 (60) | 0.105 |
| **Elevated glucose values earlier in life**  Yes  No | 440 (36)  776 (64) | 279 (36)  500 (64) | 161 (37)  276 (63) | 0.721 |
| **Family History of Diabetes**  Yes  No | 952 (78)  264 (21) | 597 (77)  182 (23) | 355 (81)  82 (19) | 0.062 |
| **Born in Europe**  Yes  No | 475 (39)  741 (61) | 198 (25)  581 (75) | 277 (63)  160 (37) | < 0.001 |
| **FINDRISC score** (median (IQR)) | 15 (13-17) | 14 (13-17) | 15 (13-18) | 0.016 ^b^ |
| ^a^ = P-value is calculated using Chi-squared tests for proportions, except those marked ^b^  ^b^ = P-value is calculated using Wilcoxon ranksum test of equality of medians | | | | |
